# DNA demethylation-mediated downregulation of *MNX1* in acute myeloid leukemia

**DOI:** 10.1038/s41375-025-02680-w

**Published:** 2025-07-14

**Authors:** Simge Kelekçi, Katherine Kelly, Ashish Goyal, Nick Wehrwein, Anna Riedel, Dieter Weichenhan, Michael Scherer, Birgitta E. Michels, Cindy Körner, Irene Orzella, Mariam Hakobyan, Marion Bähr, Elena Everatt, James Dunford, Daniel B. Lipka, Pavlo Lutsik, Udo Oppermann, Christoph Plass

**Affiliations:** 1 Division of Cancer Epigenomics, German Cancer Research Center, Heidelberg, Germany; 2 Faculty of Biosciences, Ruprecht Karl University of Heidelberg, Heidelberg, Germany; 3 Cancer-related Advanced Research and Education, TCG CREST, Kolkata, India; 4 Molecular Biosciences/Cancer Biology Programme, University of Heidelberg and German Cancer Research Centre (DKFZ), Heidelberg, Germany; 5 Division of Molecular Genome Analysis, German Cancer Research Center, Heidelberg, Germany; 6 Department of Pharmacy, Universita degli studi di Camerino, Camerino, Italy; 7 Section of Translational Cancer Epigenomics, Division of Translational Medical Oncology, DKFZ; National Center for Tumor Diseases (NCT), Heidelberg, Germany; 8 Botnar Research Centre, Nuffield Department of Orthopedics, Rheumatology and Musculoskeletal Sciences, University of Oxford, Oxford, UK; 9 Oxford Translational Myeloma Centre, University of Oxford, Oxford, UK; 10 National Center for Tumor Diseases (NCT), NCT Heidelberg, a partnership between DKFZ and Heidelberg University Hospital, Heidelberg, Germany; 11 German Cancer Consortium (DKTK), Heidelberg, Germany; 12 Department of Oncology, Catholic University Leuven, Leuven, Belgium

**Keywords:** Acute myeloid leukaemia, Cancer genomics

**To the Editor:**

Acute myeloid leukemia (AML), an aggressive hematological malignancy, is frequently associated with mutations in genes encoding enzymes that regulate epigenetic patterns. Additionally, AML is associated with genomic rearrangements, including translocations [1]. Structural rearrangements can activate oncogenes by repositioning cis-regulatory elements into the vicinity of genes, normally not expressed in a certain cell lineage, a mechanism termed enhancer hijacking [2]. For example, in the AML-M4 cell line GDM-1, the oncogenic transcription factor *Motor Neuron and Pancreas Homeobox 1* (*MNX1*) [3, 4] is aberrantly activated through enhancer hijacking due to a translocation t(6;7) which juxtaposes *MNX1* on chromosome 7 (chr7) with enhancers from the *MYB*-*AHI1* region on chr6 [3–5]. Similarly, *MNX1* activation mainly by enhancer hijacking involving various hematopoietic enhancers is a rare but recurrent event and occurs in 1.4% of primary AML [6, 7]. In this study, we hypothesized that therapeutic downregulation of *MNX1* could be achieved by an epigenetic compound disrupting the promoter-enhancer interaction. We first demonstrated with two MNX1-targeting shRNAs that *MNX1* downregulation (Supplementary Fig. 1A) reduces GDM-1 viability (Supplementary Fig. 1B). This confirms a previous finding and suggests that *MNX1* activation is required for leukemic growth [3]. To identify compounds that can reduce *MNX1* expression, we screened a focused compound library of 174 epigenetic tool compounds including approved drugs in GDM-1 cells (Supplementary Table 1a,b). Twenty-one compounds reduced viability by approximately 70% (Fig. 1A). Based on their diverse modes of action, effectiveness, and commercial availability, we selected ten compounds mediating reduced viability and determined the IC_80_ concentrations. Of these compounds, only 5-aza-2’-deoxycitidine (decitabine, DAC), a DNA methyltransferase (DNMT) inhibitor in clinical use in myelodysplastic syndrome and other hematological malignancies including AML [1], significantly suppressed *MNX1* (Fig. 1B). The reduction of viability by DAC treatment was more pronounced in GDM-1 as compared to other AML cell lines (HL-60, MOLM-13, OCI-AML-3) which do not express *MNX1* (Supplementary Fig. 1C), indicating a lower sensitivity of these lines to DAC and suggesting a positive effect of MNX1 on AML cell viability in GDM-1. In addition to reduced cell viability, DAC treatment resulted in the activation of cancer testis antigens including MAGEA3, MAGEB2, MAGEB1, FMR1NB, PAGE2B and MAEL, potentially through DNA hypomethylation. On the other hand, MNX1 transcription (Fig. 1C), and protein levels (Fig. 1D, E) were significantly reduced upon DAC treatment. This suggested the presence of a negative regulator of *MNX1* that becomes activated upon treatment. Gene ontology terms for upregulated genes were enriched for cell cycle, DNA replication & DNA repair-related processes, or tissue-specific/developmental processes, respectively (Supplementary Table 5A, B).

**Fig. 1:**
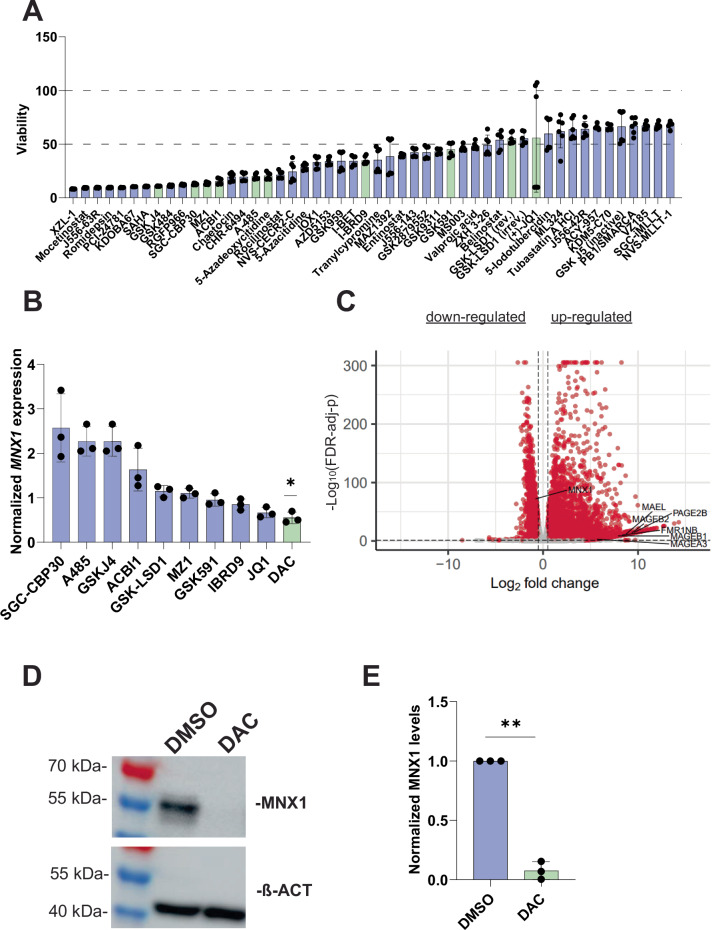
Suppression of *MNX1* expression mediated by 5-aza-2’-deoxycitidine (DAC). **A** Cell viability after treatment with the 50 most effective epigenetic compounds (refer to Supplementary Table 1). Green bars refer to compounds selected for further analysis, number 17 refers to DAC. **B** MNX1 mRNA expression was measured by qRT-PCR after treatment with ten selected compounds. The DMSO treatment was used for normalization. Quantification was performed using 2-Δ(Ct) method with GAPDH Ct-values, and the relative expression values from the compound treated and DMSO treated samples were divided by the average relative DMSO expression. These calculations were repeated for each compound. Expression after DAC treatment was reduced to 0.55-fold (Two-tailed *T* test. *p < 0.05) **C** Volcano plot showing transcriptional changes after DAC treatment. Red dots indicate significantly up- or downregulated transcripts. Genes with a false discovery rate (FDR)-adjusted *p* value (based on DESeq2) of less than 0.05 and an absolute (log2(fold change)) of greater than 1 were classified as significant. *MNX1* and cancer testis antigens are highlighted. **D** Representative Western blot of MNX1 protein levels from GDM-1 cells extracted 120 h after DAC treatment. **E** Quantification of Western Blot in D. The *p* values come from a one-tailed *t* test.

Since miRNAs are known to suppress *MNX1* [8, 9], we performed miRNA-seq on DAC-treated GDM-1 cells and DMSO controls and used three prediction tools to search for miRNAs that become upregulated and could target the *MNX1* 3ʹ untranslated region (3ʹ UTR). Six miRNAs, predicted to bind to *MNX1* 3’ UTR by at least two of three prediction tools, were upregulated upon DAC treatment (Fig. 2A). Upregulation of three miRNAs, miR-381-3p, miR-410-3p, and miR-200a-3p, which were not expressed in DMSO-treated cells, was confirmed by qRT-PCR (Supplementary Fig. 2A, Supplementary Table 2). When we expressed miRNA mimics corresponding to these three miRNAs, we observed a significant downregulation of *MNX1* only with the miR-200a-3p mimic (Fig. 2B), reaching approximately the same expression level as seen after DAC treatment. The strong reduction in MNX1 protein levels upon DAC treatment may indicate the existence of additional DAC-induced, yet unknown, miRNAs that may control MNX1 protein translation. As an indirect proof of the interaction between miR-200a-3p and the targeted *MNX1* 3’UTR and, hence, downregulation of *MNX1* by the miRNA, we quantitated fluorescence signals in HEK293T cells transfected with the miR-200a-3p mimic and luciferase constructs bearing either a wildtype or a mutated binding site of the *MNX1* 3’UTR (Supplementary Fig. 2B). We observed a significant signal reduction in cells with the wildtype but not in cells with the mutated construct (Fig. 2C). We inferred that the miR-200a-3p mimic binds to the *MNX1* 3’UTR and leads to the degradation of the luciferase transcript, suggesting that *MNX1* is downregulated by miR-200a-3p in GDM-1. Since the miR-200 family is activated by DNA demethylation of its transcription start site (TSS) in breast and bladder cancer [10, 11], we determined methylation levels in the CpG-rich TSS region of miR-200a-3p upon DAC treatment. We observed significant hypomethylation only on the 4th amplicon (Supplementary Fig. 2C), suggesting that DAC-induced promoter demethylation led to upregulation of miR-200a-3p. We also observed that overexpression of other miRNAs (miR-381-3p and miR-410-3p), which are found to be significantly upregulated upon DAC treatment, does not lead to reduction of MNX1 protein levels (Supplementary Fig. 2D).

**Fig. 2:**
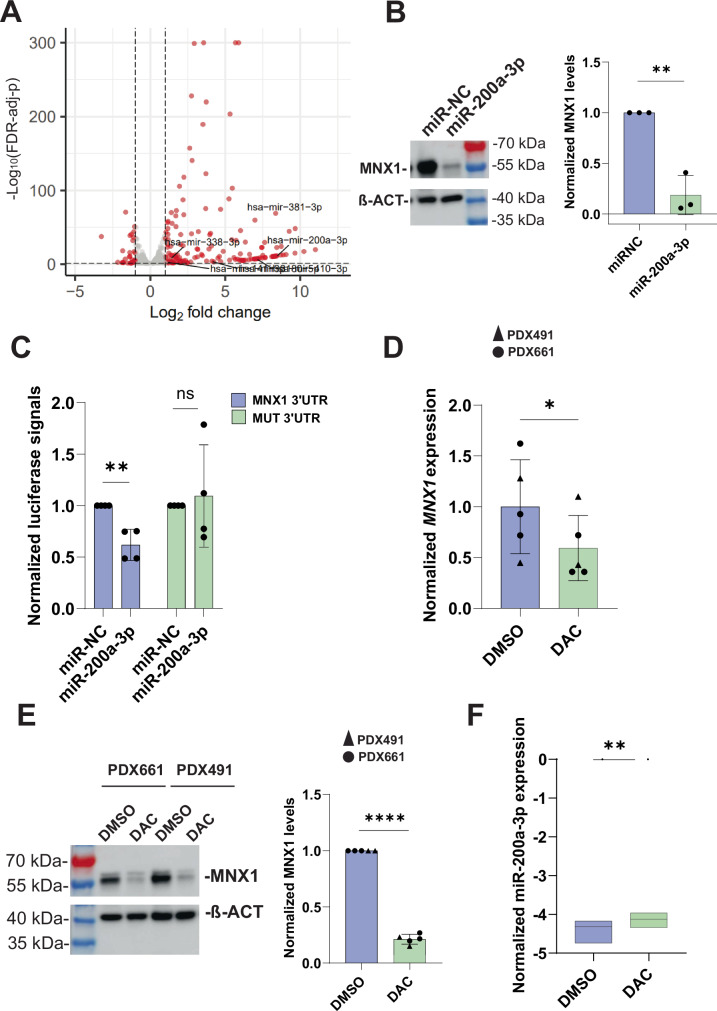
Activated miR-200a-3p suppresses *MNX1* in GDM-1 and MNX1 expressing PDX samples. **A** Volcano plot of miRNA-seq expression data from GDM-1 cells treated with DAC. Genes with a false discovery rate (FDR)-adjusted *p*-value (based on DESeq2) of less than 0.05 and an absolute (log2(fold change)) of greater than 1 were classified as significant. **B** Representative Western blot (left) and quantification (right) showing MNX1 protein expression in GDM-1 cells upon treatment with miRNA-200a-3p mimic (miRNA-200a-3p) or scrambled control (miR-NC). Each of the three biological replicates was normalized to its specific control experiment. **C** Luciferase signal intensity in HEK293 cells with mimics for miR-200a-3p and negative control (used for normalization for each of the replicated separately), NC. **D** Quantification of *MNX1* transcription in PDX491 and PDX661 after DAC treatment through qRT-PCR. The mean of the DMSO replicates was used for normalizing DAC-treated PDX samples. **E** Representative Western blot (left) and quantification (right) showing reduced MNX1 protein levels in DAC-treated PDX491 and PDX661. **F** Log2 of miR-200a-3p expression levels in DAC-treated PDX491 and PDX661. **B**–**F** The *p* values were obtained from one-tailed t-tests.

Patient-derived xenografts (PDX) in mice enable to study cancer development in vivo. Previously, we demonstrated in an AML PDX mouse model with del(7q) and MNX1 expression that shRNA-mediated knockdown of MNX1 reduces the tumor load of AML PDX cells in vivo [6]. Here, we investigated the effect of DAC treatment on *MNX1* regulation in two AML-derived PDX lines ex vivo, PDX491 and PDX661, which show aberrant *MNX1* expression due to enhancer hijacking. As in the AML cell line GDM-1, we observed a significant reduction of *MNX1* expression in both PDX lines (Fig. 2D, E). Moreover, DAC also induced upregulation of miR-200a-3p, supporting its role in *MNX1* downregulation (Fig. 2F).

In summary, we demonstrated that treatment of GDM-1 with DAC leads to miR-200a-3p activation, which, in turn, suppresses *MNX1* and reduces cell viability. Our results may have important implications for the clinical management of AML cases with ectopic *MNX1* expression including infants with t(7;12) [12] and in elderly patients with del(7q) [6]. To enforce DAC treatment and advance in *MNX1* overexpressing AML subtypes, combination therapies with other epigenetic compounds such as those that might disrupt the enhancer-promoter interaction activating *MNX1* are warranted. Possible candidates are bromodomain and extraterminal (BET) protein inhibitors, where multiple clinically tested chemotypes have been previously developed [13] including JQ1, which was reported to reduce oncogenic expression by interference with enhancer-promoter interaction [14, 15].

A limitation of our study is the focus on miR-200a-3p as the mechanism mediating *MNX1* downregulation upon DAC treatment. However, there may be other mechanisms at work as well. Future studies are needed to validate our findings in animal models.

## Supplementary information

Supplementary Material

## References

[1] Khwaja A, Bjorkholm M, Gale RE, Levine RL, Jordan CT, Ehninger G, et al. Acute myeloid leukaemia. Nat Rev Dis Prim. 2016;2:1–22. 10.1038/nrdp.2016.10 27159408

[2] Gröschel S, Sanders MA, Hoogenboezem R, de Wit E, Bouwman BAM, Erpelinck C, et al. A single oncogenic enhancer rearrangement causes concomitant EVI1 and GATA2 deregulation in leukemia. Cell. 2014;157:369–81. 24703711 10.1016/j.cell.2014.02.019

[3] Nagel S, Kaufmann M, Scherr M, Drexler HG, MacLeod RAF. Activation of HLXB9 by juxtaposition with MYB via formation of t(6;7)(q23;q36) in an AML-M4 cell line (GDM-1). Genes, Chromosomes Cancer. 2005;42:170–8. 15540222 10.1002/gcc.20113

[4] Waraky A, Östlund A, Nilsson T, Weichenhan D, Lutsik P, Bähr M, et al. Aberrant MNX1 expression associated with t(7;12)(q36;p13) pediatric acute myeloid leukemia induces the disease through altering histone methylation. Haematologica. 2024;109:725–39. 37317878 10.3324/haematol.2022.282255 PMC10905087

[5] Weichenhan D, Riedel A, Meinen C, Basic A, Toth R, Bähr M, et al. Translocation t(6;7) in AML-M4 cell line GDM-1 results in MNX1 activation through enhancer-hijacking. Leukemia. 2023;37:1147–50. 36949154 10.1038/s41375-023-01865-5 PMC10169647

[6] Sollier E, Riedel A, Toprak UH, Wierzbinska JA, Weichenhan D, Schmid JP, et al. Enhancer hijacking discovery in acute myeloid leukemia by pyjacker identifies MNX1 activation via deletion 7q. Blood Cancer Discov. 2025;6:343–63. 10.1158/2643-3230.BCD-24-0278 PMC12209774 40162972

[7] Weichenhan D, Riedel A, Sollier E, Toprak UH, Hey J, Breuer K, et al. Altered enhancer-promoter interaction leads to MNX1 expression in pediatric acute myeloid leukemia with t(7;12)(q36;p13). Blood Adv. 2024;8:5100–11. 39121370 10.1182/bloodadvances.2023012161 PMC11460460

[8] Chen H, Zeng L, Zheng W, Li X, Lin B. Increased expression of microRNA-141-3p improves necrotizing enterocolitis of neonates through targeting MNX1. Front Pediatr. 2020;8:555135. 10.3389/fped.2020.00385 PMC7399201 32850524

[9] Mu C, Wang T, Wang X, Tian H, Liu Y. Identification of microRNAs regulating Hlxb9 gene expression during the induction of insulin-producing cells. Cell Biol Int. 2016;40:515–23. 26801823 10.1002/cbin.10586

[10] Neves R, Scheel C, Weinhold S, Honisch E, Iwaniuk KM, Trompeter H-I, et al. Role of DNA methylation in miR-200c/141 cluster silencing in invasive breast cancer cells. BMC Res Notes. 2010;3:219. 20682048 10.1186/1756-0500-3-219 PMC3161370

[11] Wiklund ED, Bramsen JB, Hulf T, Dyrskjøt L, Ramanathan R, Hansen TB, et al. Coordinated epigenetic repression of the miR-200 family and miR-205 in invasive bladder cancer. Int J Cancer. 2011;128:1327–34. 20473948 10.1002/ijc.25461

[12] Espersen ADL, Noren-Nyström U, Abrahamsson J, Ha S-Y, Pronk CJ, Jahnukainen K, et al. Acute myeloid leukemia (AML) with t(7;12)(q36;p13) is associated with infancy and trisomy 19: Data from Nordic Society for Pediatric Hematology and Oncology (NOPHO-AML) and review of the literature. Genes Chromosomes Cancer. 2018;57:359–65. 29569294 10.1002/gcc.22538

[13] Wu Q, Heidenreich D, Zhou S, Ackloo S, Krämer A, Nakka K, et al. A chemical toolbox for the study of bromodomains and epigenetic signaling. Nat Commun. 2019;10:1–14. 31015424 10.1038/s41467-019-09672-2 PMC6478789

[14] Lovén J, Hoke HA, Lin CY, Lau A, Orlando DA, Vakoc CR, et al. Selective inhibition of tumor oncogenes by disruption of super-enhancers. Cell. 2013;153:320–34. 23582323 10.1016/j.cell.2013.03.036 PMC3760967

[15] Wang J, Huang TY-T, Hou Y, Bartom E, Lu X, Shilatifard A, et al. Epigenomic landscape and 3D genome structure in pediatric high-grade glioma. Sci Adv. 2021;7:eabg4126. 34078608 10.1126/sciadv.abg4126 PMC10166578

