# Supplementary material for: DNA demethylation-mediated downregulation of MNX1 in acute myeloid leukemia
Source: Leukemia. 2025 Jul 14;39(9):2270–4. doi: 10.1038/s41375-025-02680-w (PMC12380614; doi:10.1038/s41375-025-02680-w)
Supplement: Supplementary file 1 — Supplementary Material [file 41375_2025_2680_MOESM1_ESM.pdf]

Supplemental Material:

DNA DEMETHYLATION-MEDIATED DOWNREGULATION OF *MNX1* IN ACUTE MYELOID LEUKEMIA

Simge Kelekçi,[1,2], Katherine Kelly[1,2], Ashish Goyal[1,3], Nick Wehrwein[1,4], Anna Riedel[1,2], Dieter Weichenhan[1], Michael Scherer[1], Birgitta E. Michels[5], Cindy Körner[5], Irene Orzella[6], Mariam Hakobyan[7], Marion Bähr[1], Elena Everatt[1,2], James Dunford[8,9], Daniel B. Lipka[7,10,11], Pavlo Lutsik[1,12], Udo Oppermann[8,9], Christoph Plass[1]

METHODS

Cell lines and PDX cultivation

OCI-AML-3 (CVCL_1844), HL-60 (CVCL_0002) and MOLM-13 (CVCL_2119) were cultured in RPMI 1640 (Gibco, 21875-034) supplemented with 10% fetal bovine serum (FBS; Sigma Aldrich, S0615) and 100U/mL penicillin/streptomycin (P/S, Sigma Aldrich, 10.000 units, P0781). GDM-1 (CVCL_1230) was cultured in RPMI 1640 supplemented with 20% FBS. HEK293T (CVCL_0063) was cultured in Dulbecco's modified Eagle's medium (DMEM) 4.5 g/L D-glucose, L-glutamine (-) Pyruvate (Thermo Fischer, 41965) supplemented with 10% fetal bovine serum and 100U/mL penicillin/streptomycin (P/S, Sigma Aldrich, 10.000 units, P0781). Cell line authentication was performed in March (GDM-1) and December 2023 (OCIAML3, HL60, MOLM13), and all cell lines are regularly tested for mycoplasma contamination using a commercial test kit (VenorGeM Classic, Minerva Biolabs, cat.no. 11-1050, Berlin).

Animal experiments were carried out following the current ethical standards of the Committee on Animal Experimentation (Government of Upper Bavaria, tierversuche@reg-ob.bayern.de; ROB-55.2Vet-2532.Vet_02-16-7 and ROB-55.2-2532.Vet_02-20-159). PDX491 and PDX661 were generated from first and second-relapse of a del(7q) patient with *MNX1* expression [1,2]. Mouse cells were depleted before cultivation using the Mouse Cell Depletion Kit (Miltenyi Biotec, 130-140-694). PDX491 and PDX661 were cultured in StemPro-34 Medium (Thermo Fischer, 10639011) supplemented with 100U/mL penicillin/streptomycin (P/S, Sigma Aldrich, 10.000 units, P0781), 0.1% of 50mg/ml Gentamicin (Lonza, 17-518L), 1% of 200 mM L-Glutamin (Gibco, 25030-024), 10ng/ml of rhFLT3 (R&D Systems, 308-FKN-100), 10 ng/ml of rhSCF (Peprotech, 300-07-100), rhTPO (Peprotech, 300-18-100) and rhIL3 (Peprotech, 200-03-100).

Epigenetic compound screening and treatment regimen

35000 GDM-1 cells/well were seeded 4 hours before the treatment with the epigenetic compound library [3] (Supplementary Table 1). Cell viability was assessed via CellTiter-Blue cell viability assay (Promega, G9081) after 120 hours and normalized to DMSO-treated samples. For IC50 and IC80 determination after 120 hours, compounds with varying concentrations were applied individually. IC50 and IC80 were calculated using non-linear regression (curve fit) with GraphPad Prism 10.2.2.

In specific experiments with 5-aza-2'-deoxycitidine (DAC) treatment, 500 nM of DAC was applied (Sigma, cat.no.:A3656) for 120 hours. DAC-containing media were refreshed every 24 hours.

Cre-ER mediated shRNA and GFP expression were activated by addition of 1 µg/mL 4-Hyrodxytamoxifen (Sigma, cat.no: 508225). BFP was used to sort the transfected cells, and GFP was used to sort the cells with active shRNA. 48 hours after the treatment, GFP- and BFP-expressing cells were sorted using fluorescence-activated cell sorting (FACS), cultured for cell viability assay, or pelleted for further analysis.

RNA isolation and quantitative real-time PCR (qRT-PCR)

Total RNA was isolated using the Qiagen RNeasy Plus Mini kit (Qiagen, cat.no.: 74136), and quantified using a Qubit kit according to the manufacturer's instructions (Invitrogen, cat.no.: Q32855). cDNA was synthesized using random hexamers and Superscript III reverse transcriptase (Invitrogen, cat.no.: 56575). QRT-PCR (primers in **Supplementary Table 3**) was performed with the Roche Lightcycler 480 using a primaQUANT CYBR mix (Steinbrenner Laborsyteme GmbH, cat.no.: SL-9902). Quantification was performed using the $2^{-\Delta(Ct)}$ method with *GAPDH* Ct-values used for normalization.

RNA sequencing analysis

RNA was sequenced on NOVASEQ 6000 with mode 100 bp paired end S1. The data were processed using the nf-core RNA-seq pipeline, which involved quality control with fastqc, UMI barcode extraction using UMI tools, adapter and quality trimming using TrimGalore, read alignment (hg19), and quantification using Salmon/STAR [4]. Differential gene expression before and after DAC treatment was assessed using DESeq2 [5] with R version 4.1.0. Genes with false discovery rate (FDR)-adjusted p-value < 0.05 and absolute log2 fold change > 0.5 were considered differentially expressed. Enrichment analysis for Gene Ontology biological processes was performed using the ClusterProfiler R package. Processes with Benjamini-Hochberg-adjusted p-value < 0.05 were considered significantly enriched.

Protein Isolation and Western blot

Cells were washed twice with DPBS (Gibco, cat.no.: 14190094) and lysed for 30 minutes on ice, followed by 10 minutes at 97°C in hot-lysis buffer composed of 62.5 mM Tris-HCl pH 6.8, 2% sodium dodecyl sulfate (SDS), 10% glycerol, 1 mM DTT, 1 mM NaVO4, 5 mM NaF supplemented with one tablet of PhosStop (Roche, cat.no. 4906837001), and one tablet of

complete protease inhibitor (Roche, cat.no. 11697498001, Mannheim, Germany) and 25 U/mL Benzonase (Santa Cruz Biotechnology, cat.no. sc-202391, Heidelberg, Germany). Lysates were cleared by centrifugation, protein concentrations were determined using the BCA assay (Sigma, cat.no.: B9643-1L). Per lane of a 4-20% SDS-PAGE gradient gel (BioRad, cat.no. 4561096, Dreieich, Germany), 20 µg protein were separated and transferred onto a polyvinylidene difluoride (PVDF) membrane (Millipore, cat.no. IPVH00010, Darmstadt, Germany). The membrane was probed with a primary antibody against MNX1 (Bethyl, cat.no A303-184A-T; RRID:) in a dilution of 1:1000 and for loading control with an antibody against β-actin (Santa Cruz Biotechnology, cat.no. sc-47778; RRID:). The protein signal was visualized after the incubation with the Novex ECL HRP Chemiluminescent Substrate Reagent Kit (Invitrogen Thermo Fisher Scientific, cat.no. WP20005) using the imager Amersham 680 (GE Healthcare). Band intensities were quantitated with Image Studio Version 5.2.

miRNA mimic-dependent regulation in GDM-1

GDM-1 cells were electroporated with miRNA mimics (hsa-miR-200a-3p, hsa-miR-410-3p, hsa-miR-381-3p) (Thermo Fischer, miScript mimics) using the Neon Transfection System (Thermo Fischer, cat. no. MPK1025) at 1,500 V, 20 ms, and 1 pulse. MiRNA isolation was performed using miRNeasy Micro Kit (Qiagen, cat.no. 217084) and was quantified with a Qubit kit according to manufacturer's instructions (Invitrogen, cat.no.: Q32855). Complementary DNA (cDNA) was synthesized using miRCURY LNA RT kit (Qiagen, cat. no. 339340). QRT-PCR was performed using the miRCURY LNA SYBR Green PCR Kit (Qiagen, cat. no. 339346) (probes in **Supplementary Table 4**). Quantification was performed using the $2^{-\Delta(Ct)}$ method with *SNORD44* Ct-values used for normalization.

Small-RNA sequencing library preparation

MiRNA isolation was performed using the miRNeasy Micro Kit (Qiagen, cat.no. 217084). MiRNA-seq libraries were prepared using the NEBNext Small RNA Library Prep Set for Illumina (NEB, cat.no. E7300S) and purified using Monarch PCR and DNA cleanup kit (NEB, cat.no. T1030S). Fragments were purified with AMPure XP Beads (Beckman coulter, cat. no. A63880) in 1:1,3 and subsequent 1:3,7 ratio. Library concentrations were measured using the Qubit HS DNA kit (Invitrogen, cat.no.: Q32853). Library quality was assessed using a TapeStation 4150 with D100 High Sensitivity Assay (Agilent, cat.no.: 11691112001). Libraries were sequenced with NextSeq 550, single-read 75 bp high-output mode.

Small-RNA sequencing data analysis

Reads were aligned to hg38 using the bowtie aligner in sRNAmapper, accessed via the Heidelberg Unix Sequence Analysis Resources (HUSAR) GUI. Resulting bam files were indexed using samtools. The data were filtered to remove miRNA primary transcripts and sequences encoded from multiple genomic locations and those lowly expressed across samples (< 5 median raw read counts across samples). MiRNA-level and arm-level counts were computed from the raw read counts and used for differential expression analysis. Differential expression of miRNAs before and

after DAC treatment was evaluated using DESeq2 with R version 4.1.0. MiRNAs predicted to target the MNX1 3' UTR were retrieved from three miRNA target prediction databases: miRDB, TargetScan, and miRTarBase. MiRNAs that were predicted to target MNX1 by at least two of the three prediction tools and were significantly upregulated by DAC treatment after correction for multiple testing (Benjamini Hochberg method) were selected for further investigation.

Luciferase assay

6.3 x 10^5 HEK293T cells were transfected with 5 nM of miRNA mimics using DharmaFect Transfection reagent (Dharmacon, cat. no. T-2001-03) in one well of a 12-well plate. 24 hours after transfection, cells were transfected with 1 µg of plasmid psiCHECK2.0 with a wildtype (ENST00000252971.6, chr7:156,797,547-156,798,213) or a mutated *MNX1* 3'UTR (gene synthesized and cloned by BioCat, Heidelberg) using TransIT-LT1 (Mirus, cat.no. MIR2304). Cells were harvested 48 hours after the transfection, seeded onto 384-well plates and analyzed with the Dual-Glo Luciferase Assay System (Promega, cat.no. E2920) following the manufacturer's instructions.

Local Deep Bisulfite Sequencing

Bisulfite treatment of 500 ng genomic DNA (gDNA) before and after DAC treatment was performed using the EZ Methylation kit (Zymo Research, cat.no. D5001). 1.5 µl of bisulfite-treated gDNA eluate were mixed with 3.7 µl of H_2O, 0.7 µl of 10X Taq polymerase buffer (Qiagen, cat.no.: 1005479), 0.056 µl of Hot Start Taq Polymerase (Qiagen, cat.no.: 1007837), 1 µM of forward and reverse primer mix (**Supplementary Table 3**) and 0.056 µl of 10 mM dNTP (Thermo Fischer, cat.no.: 0192). Reaction mixes were incubated at 94°C for 15 minutes, followed by 45 cycles of 95°C for 30 seconds, 62°C, 72°C for 60 seconds, and finally at 72°C for 5 minutes. A second PCR was performed under real-time conditions using 0.5 ng of the first PCR product, 9.75 µl H2O, 0.25 µl of primaQUANT CYBR mix (Steinbrenner Laborsyteme GmbH, cat.no.: SL-9902), 12.5 µl of 2X Kappa 2G Robust Hotstart Readymix (Kapa Biosystems, cat.no.: KK5702), 0.75 µl of 10µM Tn5McP1n and 0.75 µl of 10µM barcode primers. Samples were incubated at 98°C for 30 seconds, followed by eight cycles of 98°C for 10 seconds, 63°C for 10 seconds, 72°C for 10 seconds. PCR products were purified using Ampure beads with a 1:1.4 beads-to-DNA ratio. Library concentration and amplicon size were determined with the Qubit dsDNA HS kit (Invitrogen, cat.no.: Q32853) and the High Sensitivity D1000 Tapestation kit (Agilent, cat.no.: 5067-5585), respectively. Samples from different amplicons were multiplexed in equal molar ratios and submitted to the MiSeq Nano V2 (4-color) platform. Tabsat was used to analyze bisulfite amplicon sequencing data [6]. Briefly, raw sequencing reads were aligned to hg19 using Bismark [7]. The number of methylated/unmethylated cytosines for each amplicon was then computed and returned in tabular form. Average DNA methylation values across all the sequencing reads for each CpG covered by the amplicons were visualized using Figeno [8].

Statistical analysis

Unless otherwise stated, an unpaired, one-tailed Student's t-test was used to analyze the data from at least three corresponding replicates using GraphPad PRISM 10.2.2. We opted for the one-tailed

test, since we specifically tested for downregulation of MNX1 upon DAC treatment. In the initial test shown in Figure 1B, a two-tailed Student's t-test was used, since we tested both for up- and downregulation at the same time. Mean ± standard deviation (s.d.) was used as a precision measure. Data were considered statistically significant with thresholds $p < 0.05$ (*), $p \leq 0.01$ (**), $p \leq 0.001$ (***), $p \leq 0.0001$ (****).

References:

1 Sollier E, Riedel A, Toprak UH, Wierzbinska JA, Weichenhan D, Schmid JP *et al.* Enhancer hijacking discovery in acute myeloid leukemia by pyjacker identifies MNX1 activation via deletion 7q. *Blood Cancer Discov* 2025. doi:10.1158/2643-3230.BCD-24-0278.

2 Zeller C, Richter D, Jurinovic V, Valtierra-Gutiérrez IA, Jayavelu AK, Mann M *et al.* Adverse stem cell clones within a single patient's tumor predict clinical outcome in AML patients. *J Hematol Oncol* 2022; **15**: 25.

3 Cottone L, Cribbs AP, Khandelwal G, Wells G, Ligammari L, Philpott M *et al.* Inhibition of Histone H3K27 Demethylases Inactivates Brachyury (TBXT) and Promotes Chordoma Cell Death. *Cancer Res* 2020; **80**: 4540–4551.

4 Dobin A, Davis CA, Schlesinger F, Drenkow J, Zaleski C, Jha S *et al.* STAR: ultrafast universal RNA-seq aligner. *Bioinformatics* 2013; **29**: 15–21.

5 Love MI, Huber W, Anders S. Moderated estimation of fold change and dispersion for RNA-seq data with DESeq2. *Genome Biol* 2014; **15**: 550.

6 Pabinger S, Ernst K, Pulverer W, Kallmeyer R, Valdes AM, Metrustry S *et al.* Analysis and Visualization Tool for Targeted Amplicon Bisulfite Sequencing on Ion Torrent Sequencers. *PLoS One* 2016; **11**: e0160227.

7 Krueger F, Andrews SR. Bismark: a flexible aligner and methylation caller for Bisulfite-Seq applications. *Bioinformatics* 2011; **27**: 1571–1572.

8 Sollier E, Heilmann J, Gerhauser C, Scherer M, Plass C, Lutsik P. Figeno: multi-region genomic figures with long-read support. *Bioinformatics* 2024; **40,** 3;40(6):btae354.

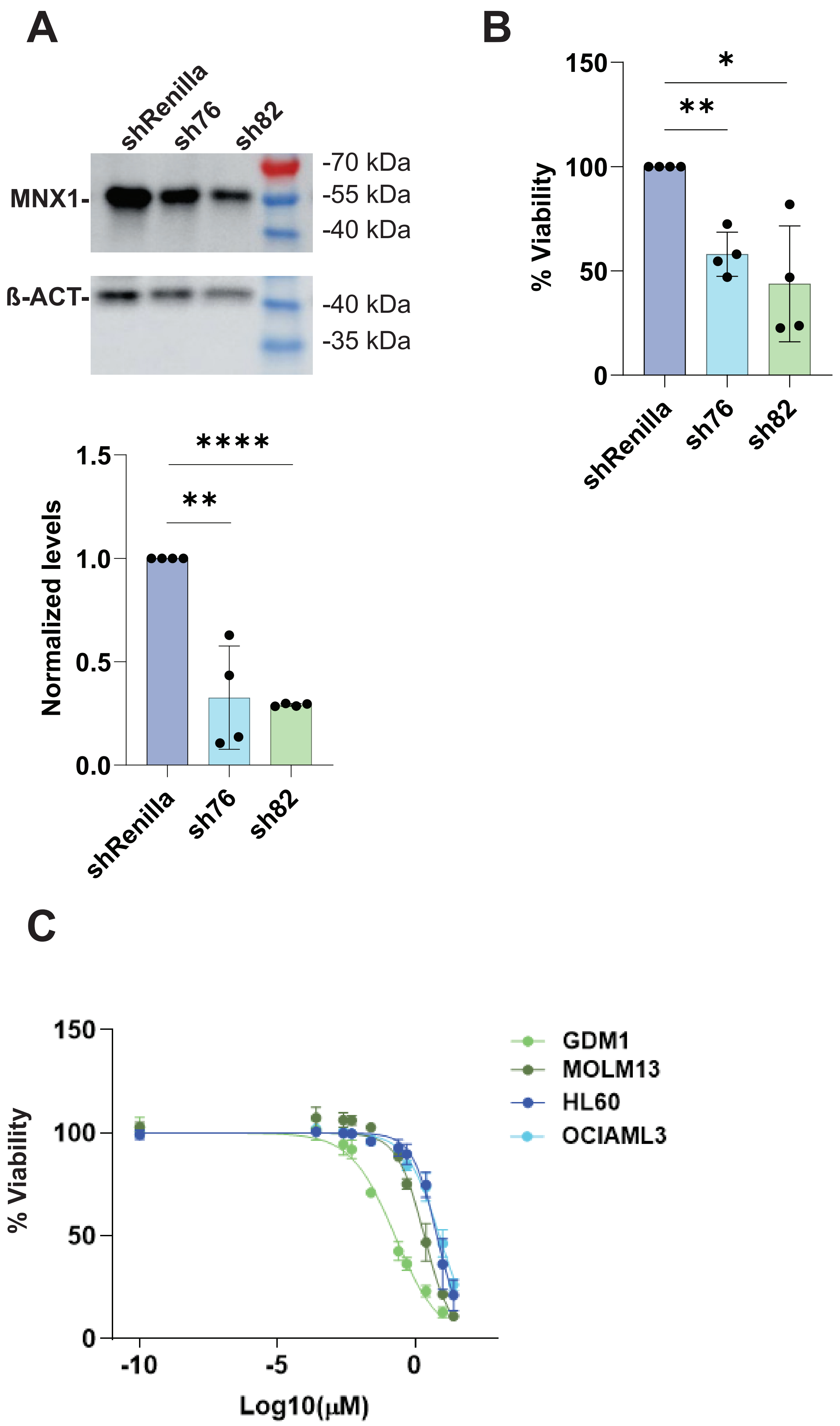
A
shRenilla
sh76
sh82
MNX1-
ß-ACT-
-70 kDa
-55 kDa
-40 kDa
-40 kDa
-35 kDa
Normalized levels
1.5
1.0
0.5
0.0
****
**
B
% Viability
150
100
50
0
*
**
C
GDM1
MOLM13
HL60
OCIAML3
-10
-5
0
Log10(µM)

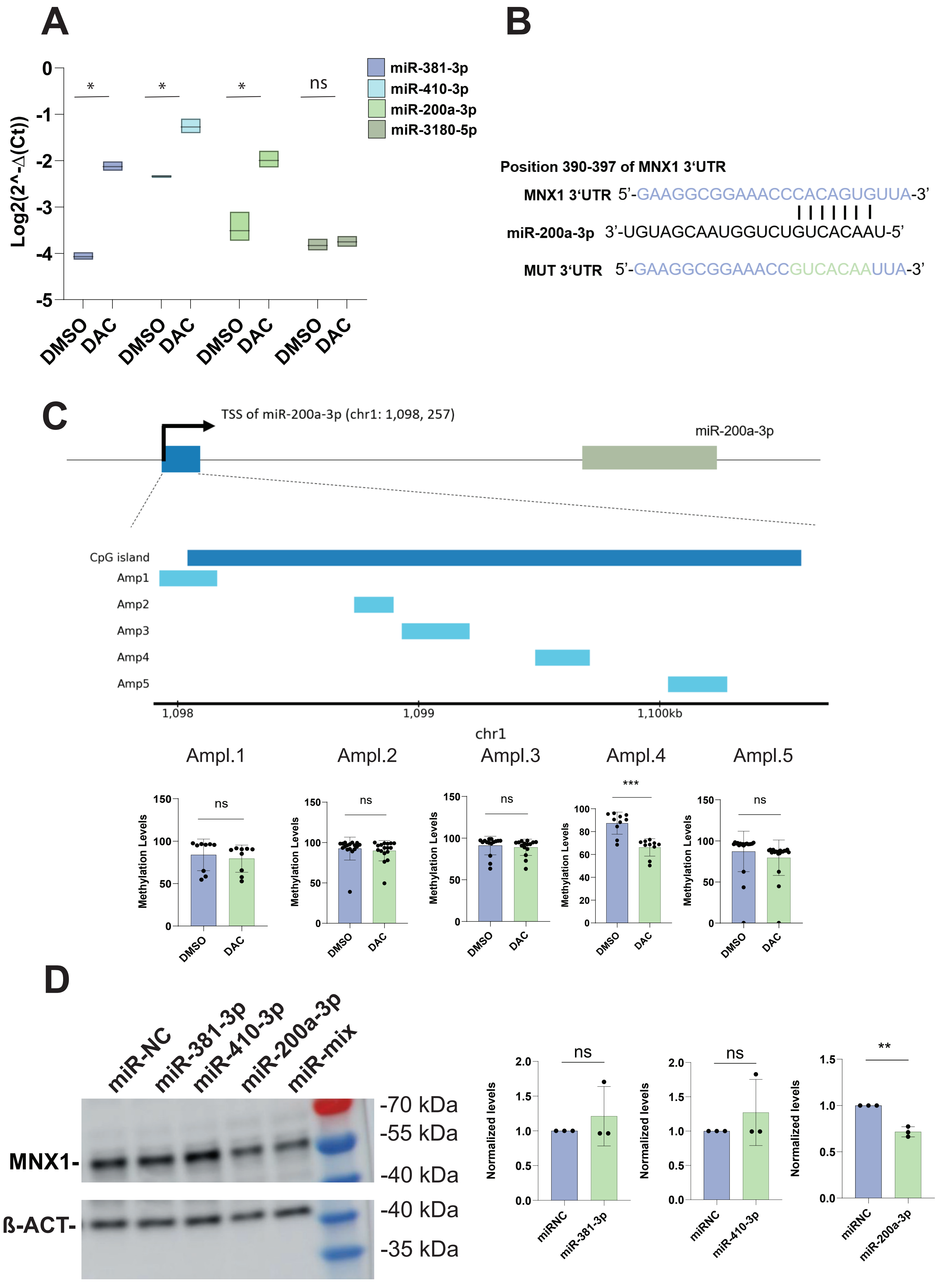
A
Log2(2^-Δ(Ct))
0
-1
-2
-3
-4
-5
*
*
*
ns
DMSO
DAC
miR-381-3p
miR-410-3p
miR-200a-3p
miR-3180-5p
B
Position 390-397 of MNX1 3'UTR
MNX1 3'UTR 5'-GAAGGCGGAAACCCACAGUGUUA-3'
miR-200a-3p 3'-UGUAGCAAUGGUCUGUCACAAU-5'
MUT 3'UTR 5'-GAAGGCGGAAACCGUCACAAUUA-3'
C
TSS of miR-200a-3p (chr1: 1,098, 257)
miR-200a-3p
CpG island
Amp1
Amp2
Amp3
Amp4
Amp5
1,098
1,099
1,100kb
chr1
Ampl.1
Ampl.2
Ampl.3
Ampl.4
Ampl.5
Methylation Levels
ns
***
DMSO
DAC
D
miR-NC
miR-381-3p
miR-410-3p
miR-200a-3p
miR-mix
-70 kDa
-55 kDa
-40 kDa
-40 kDa
-35 kDa
MNX1-
ß-ACT-
Normalized levels
ns
**
miRNC
miR-381-3p
miR-410-3p
miR-200a-3p

SUPPLEMENTARY TABLES

Supplementary Table1a. compounds 1-50 and their doses (Figure 1A)

**Compound number**	**Name**	**Doses (µM)**
1	XZL-1	1
2	Mocetinostat	10
3	J556-63R	1
4	Romidepsin	1
5	PCI-24781	5
6	KDOBA67	10
7	SAHA	2.5
8	GSK J4	10
9	GSK484	1
10	RGFP966	10
11	SGC-CBP30	1
12	MZ1	1
13	ACBI1	1
14	Chaetocin	0.05
15	CHR-6494	1
16	A-485	1
17	5-Azadeoxycitidine	5
18	Rocilinostat	10
19	NVS-CECR2-C	1
20	5-Azacitidine	10
21	IOX1	40
22	AZD5153	1
23	GSK959	1
24	I-BET	1
25	I-BRD9	10
26	Tranylcypromine	10
27	MAZ1392	1
28	Entinostat	0.5
29	J556-143	1
30	GSK2879552	10
31	GSK9311	1
32	GSK591	1
33	MS003	1
34	Valproic acid	1000
35	ZXH 3-26	0.1
36	Belinostat	5
37	GSK690 /GSK-LSD1 (reversible)	5
38	GSK-LSD1 (irreversible)	0.5
39	(+)-JQ1	1
40	5-Iodotubercidin	1
41	ML324	5
42	Tubastatin A HCl	10
43	J556-42R	1
44	ACY-957	1
45	KDM5-C70	10
46	GSK J5 (inactive)	10
47	PB1/SMARCA	1
48	VZ185	1
49	SGC-iMLLT	1
50	NVS-MLLT-1	1

Supplementary Table1b. All compounds used in the screening process.

Source Name	Class/Target
(+)-JQ1	Bromodomains - BRD2, BRD3, BRD4, BRDT (BET)
(-)-JQ1	Bromodomains - (Neg Ctrl)
PFI-1	Bromodomains - BRD2, BRD3, BRD4, BRDT (BET)
I-BET	Bromodomains - BRD2/3/4
Bromosporine	Bromodomains - pan-Bromodomain
CBP/BRD4 (0383)	Bromodomains - CBP, BRD4(1)
SGC-CBP30	Bromodomains - CREBBP, EP300
I-CBP112	Bromodomains - CREBBP, EP300
RVX-208	Bromodomains - BRD2,3,4, BRDT (BET, BD2)
SMARCA	Bromodomains - SMARCA, PB1
PB1/SMARCA	Bromodomains - SMARCA, PB1
PFI-3	Bromodomains - SMARCA2/4, PB1(5)
GSK2801	Bromodomains - BAZ2A, BAZ2B
PFI-4	Bromodomains - BRPF1B
TRIM24/BRPF	Bromodomains - TRIM24/BRPF
dTRIM24	Bromodomains - Trim24
IACS9571	Bromodomains - Trim24
OF-1	Bromodomains - pan-BRPF
BAZ2-ICR	Bromodomains - BAZ2A, BAZ2B
NI-57	Bromodomains - pan-BRPF
LP99	Bromodomains - BRD9, BRD7
BI-9564	Bromodomains - BRD9, BRD7
NVS-CECR2-1	Bromodomains - CECR2
NVS-CECR2-C	Bromodomains - CECR2 (Neg Ctrl)
GSK8814	Bromodomains - ATAD2
GSK8815	Bromodomains - ATAD2 (Neg Ctrl)
GSK959	Bromodomains - BRPF1
BAY-299	Bromodomains - BRD1, TAF1
I-BRD9	Bromodomains - BRD9
TP-472	Bromodomains - BRD9
TP-472N	Bromodomains - BRD9
GSK6853	Bromodomains - BRPF1/2/3
GSK9311	Bromodomains - BRPF1/2/3
GSK4027	Bromodomains - PCAF, GCN5
GSK4028	Bromodomains - PCAF, GCN5
L-Moses	Bromodomains - PCAF, GCN5
D-Moses	Bromodomains - PCAF, GCN5
dBRD9	Bromodomains - BRD9
BI-7273	Bromodomains - BRD9/7
BI9564	Bromodomains - BRD9
BI6354	Bromodomains - BRD9 (Neg Ctrl)
MZ1	Bromodomains - BET Protac
cisMZ1	Bromodomains - BET Protac (Neg Ctrl)
NVS-BPTF-C	Bromodomains - BPTF- (Neg Ctrl)
NVS-BPTF-1	Bromodomains - BPTF
TP422	Bromodomains - (neg ctrl)
TP238	Bromodomains - CECR2, BPTF (FALZ)
ZXH 3-26	Bromodomains - BRD4 degrader
ACBI1	Bromodomains - SMARCA2/4 PROTAC
cisACBI1	Bromodomains - SMARCA2/4 PROTAC (Neg Ctrl)
VZ185	Bromodomains - BRD7, BRD9 PROTAC
cis VZ185	Bromodomains - BRD7, BRD9 PROTAC (Neg Ctrl)
AZD5153	Bromodomains - BRD2/4
CPI-360	Histone methyltransferase - EZH2 and EZH1
UNC0638	Histone methyltransferase - G9a, GLP
UNC0642	Histone methyltransferase - G9a, GLP
A-366	Histone methyltransferase - G9a, GLP
Chaetocin	Histone methyltransferase - SUV39H1
PFI-2	Histone methyltransferase - SETD7
SGC0946	Histone methyltransferase - DOT1L
GSK343	Histone methyltransferase - EZH2
UNC1999	Histone methyltransferase - EZH2
LLY-507	Histone methyltransferase - SMYD2
A-196	Histone methyltransferase - SUV420H1/H2
BAY-598	Histone methyltransferase - SMYD2
PFI-5	Histone methyltransferase - SMYD2
CPI-169	Histone methyltransferase - EZH2, EZH1

UNC2400	Histone methyltransferase - EZH2
PFI-5NC	Histone methyltransferase - SMYD2 (Neg Ctrl)
BAY6035	Histone methyltransferase - SMYD3
Tazemetostat (EPZ-6438)	Histone methyltransferase - EZH2
XZL-1	Lysine demethylases - KDM1
GSK2879552	Lysine demethylases - LSD1
CPI-621	Lysine demethylases - KDM5
Tranylcypromine	Lysine demethylases - LSD1
GSK-LSD1 (irreversible)	Lysine demethylases - LSD1
GSK690	Lysine demethylases - LSD1
GSK J4	Lysine demethylases - JMJD3, UTX, JARID1B
GSK J5	Lysine demethylases - (Neg Ctrl)
IOX1 (5-carboxy-8HQ)	Lysine demethylases - pan-2-OG
Methylstat (Ester)	Histone demethylase
(E)-JIB-04	Histone demethylase - Pan JmjC
ML324	Histone demethylase - JMJD2E
KDOBA67	Histone demethylase - KDM6
KDOAM-25a	Lysine demethylases - JARID
KDM5-C70	Histone demethylase - JARID1
KDOAM32	Lysine demethylases - JARID
KDOPZ-32a	Lysine demethylases - KDM5
KDOOA012000	Lysine demethylases - KDM2
IOX2	Prolyl-Hydroxylases - PHD2 (EGLN1)
5-Azacitidine	DNA methyltransferase - (DNMT) -
5-Azadeoxycitidine	DNA methyltransferase - (DNMT) - DNMT1/3
Olaparib	Poly ADP ribose polymerase (PARP)
Rucaparib	Poly ADP ribose polymerase (PARP)
DUAL1946	
GSK484	Peptidyl arginine deiminase (PAD4)
GSK106	Peptidyl arginine deiminase (PAD4)
MAZ1805	Prolyl tRNA synthetase - less active
MAZ1392	Prolyl tRNA synthetase
SGI-1776	Kinase inhibitor - Haspin
CHR-6494	Kinase inhibitor - Haspin
K00135	Kinase inhibitor - ATP competitive - PIM
5-Iodotubercidin	Kinase inhibitor - ATP mimetic - Haspin
BI1347	Kinase inhibitor - Kinase CDK8
BI1374	Kinase inhibitor - Kinase CDK8 (Neg Ctrl)
GSK864	Dehydrogenase
CPTH2	HAT inhibitor - GCN5, KAT3B
TTK21	HAT activator
WM-1119	lsine acetyltransferase - KAT6A/MOZ
MOZ-IN-3	lsine acetyltransferase KAT6A/MOZ
A-485	Histone acetyltransferase (HAT) - p300/CBP
A-486	Histone acetyltransferase (HAT) - p300/CBP
C646	Histone acetyltransferase (HAT) - p300/CBP
A-395	Methyl Lysine Binder - EED
A-395N	Methyl Lysine Binder - EED
OICR-9429	Methyl Lysine Binder - WDR5
UNC1215	Methyl Lysine Binder - L3MBTL3
YX39-31b	Methyl Lysine Binder/tudor domain - Spin1
VinSpinIC	Methyl Lysine Binder/tudor domain - Spin1
VinSpinIn	Methyl Lysine Binder/tudor domain - Spin1
YX116-56B	Methyl Lysine Binder/tudor domain - Spin1
YX49-92B	Methyl Lysine Binder/tudor domain - Spin1
YX85-35	Methyl Lysine Binder/tudor domain - Spin1
UNC3866	Methyl lysine binder - CBX7/CBX4
UNC4219	Methyl lysine binder - CBX7/CBX4 (Neg Ctrl)
UNC6934	Methyl lysine binder - NSD2-PWWP1

UNC7145	Methyl lysine binder - NSD2-PWWP1 (Neg Ctrl)
BI9321	Methyl lysine binder - NSD3 (PWWP1)
BI9466	Methyl lysine binder - NSD3 (PWWP1) (Neg Ctrl)
SGC-iMLLT	Chromatin reader - MLLT1
NVS-MLLT-1	Chromatin reader - MLLT1
RTS-V5	protesome and HDAC
J556-42R	Arginine methyltransferase - PRMT5
J556-63R	Arginine methyltransferase - PRMT5
J556-70R	Arginine methyltransferase - PRMT5
MS409N	Arginine methyltransferase - PRMT4, PRMT6 (Neg Ctrl)
TP-064	Arginine methyltransferase - PRMT4
TP-064N	Arginine methyltransferase - PRMT4
LLY-283	Arginine methyltransferase - PRMT5
Ski73	Arginine methyl transferase - PRMT4 (CARM1)
SGC6870	Arginine methyl transferase - PRMT6
SGC6870N	Arginine methyl transferase - PRMT6 (Neg Ctrl)
J556-143	Arginine methyltransferase - PRMT5
MS049	Arginine methyltransferase
MS023	Arginine methyltransferase - Type I PRMTs
MS003	Arginine methyltransferase - (Neg Ctrl)
AMI-1	Arginine methyltransferase - PRMT
SGC707	Arginine methyltransferase - PRMT3
GSK591	Arginine methyl transferase - PRMT5
SGC3027	Arginine methyl transferase - PRMT7
SGC3027N	Arginine methyl transferase - PRMT7
MRK-740-NC	Arginine methyl transferase - PRDM9 (Neg Ctrl)
Belinostat	HDAC - hydroxamic acids
CXD101	HDAC -
Valproic acid	HDAC - aliphatic acid compounds
Entinostat	HDAC - ortho-amino anilides
SAHA	HDAC - hydroxamic acids
Trichostatin A	HDAC - hydroxamic acids - Class I & II
SRT1720	HDAC - SIRT1 (indirect?) activator
EX 527	HDAC - SIRT1
CI-994	HDAC - 1,2,3,(8)
RGFP966	HDAC - HDAC3
PCI-34051	HDAC - HDAC8
Rocilinostat	HDAC - HDAC6
Tubastatin A HCl	HDAC - HDAC6
PCI-24781	HDAC -
Romidepsin	HDAC -
Mocetinostat	HDAC -
Santacruzamate	HDAC - 2?
TMP269	HDAC -4, 5, 7 &9
AGK2	HDAC - SIRT2
TMP195	HDAC - 4,5,7,9
ACY-957	HDAC - 1/2
ACY-738	HDAC - 6

Supplementary Table 2. Significantly upregulated miRNAs predicted to bind to the *MNX1* 3’UTR

**miRNA**	**Expressed with DMSO**	**Confirmed**	**Prediction tools**
miR-381-3p	no	yes	TargetScan, miRDB
miR-410-3p	no	yes	TargetScan, miRDB
miR-200a-3p	no	yes	TargetScan, miRDB
miR-3180-5p	no	no	TargetScan, miRTarBase

miR-338-3p	yes	Not tested	TargetScan, miRTarBase
miR-141-3p	yes	Not tested	TargetScan, miRDB

Supplementary Table 3. QRT-PCR and bisulfite sequencing primers

Primer	Sequence[1]	hg19 coordinate[2]	Purpose
MNX1_F	GCCTAAGATGCCCGACTTC		qRT-PCR
MNX1_R	GGTACTTGTTGAGCTTGAACTGG		qRT-PCR
GAPDH_R	GCCCAATACGACCAAATCC		qRT-PCR
GAPDH_F	AGCCACATCGCTCAGACAC		qRT-PCR
MiR200a_CpGisF1	GGGGAGGTAGAGGTGGAGAG	chr1:1097927-1097947	bisulfite sequencing
MiR200a_CpGisR1	ACAAATATATCCCCTAAACTCCC	chr1:1098139-1098161	bisulfite sequencing
MiR200a_CpGisF2	YGTAGGTGATAGATGGGTTG	chr1:1098736-1098755	bisulfite sequencing
MiR200a_CpGisR2	RTAAATCCAAAATAACTCCACAC	chr1:1098851-1098893	bisulfite sequencing
MiR200a_CpGisF3	TAGGTATGGGGTGTTTTTAG	chr1:1098933-1098953	bisulfite sequencing
MiR200a_CpGisR3	CRACCTCTAACCAAAAAATAC	chr1:1099188-1099208	bisulfite sequencing
MiR200a_CpGisF4	TGGGAATTGATTGATTATGGTG	chr1:1099486-1099508	bisulfite sequencing
MiR200a_CpGisR4	CCAACCATACTTTTAAACATTTC	chr1:1099684-1099707	bisulfite sequencing
MiR200a_CpGisF5	YGTAGAGGGAAGAATTTGAGTG	chr1:1100038-1100060	bisulfite sequencing
MiR200a_CpGisR5	CCRAATCCCACCTACACAAAC	chr1:1100258-1100277	bisulfite sequencing

[1]Bisulfite sequencing primers share at 5'end sequence TCGTCGGCAGCGTCAGATGTGTATAAGAGACAG (forward) and sequence GTCTCGTGGGCTCGGAGATGTGTATAAGAGACAG (reverse). For secondary PCR to generate bisulfite sequencing libraries forward primers AATGATACGGCGACCACCGAGATCTACACTCGTCGGCAGCGTC and reverse primers CAAGCAGAAGACGGCATACGAGAT[barcode]GTCTCGTGGGCTCGG with an amplicon-specific barcode were used.
[2]qRT-PCR primers are in part intron spanning, hence, coordinates were omitted.

Supplementary Table 4 MiRNA probes

Probe	GeneGlobeID
miR-200a-3p qPCR	YP00204707
miR-3180-5p qPCR	YP002113327
miR-410-3p qPCR	YP00204042
miR-3180-5p qPCR	YP02113327
SNORD44 qPCR	YP00203902

Supplementary Table 5A Top 20 GO upregulated pathways

	Description	qvalue
GO:0006260	DNA replication	2.89E-52

GO:0006261	DNA-dependent DNA replication	9.91E-46
GO:0140053	mitochondrial gene expression	2.42E-45
GO:0007059	chromosome segregation	7.78E-43
GO:0032543	mitochondrial translation	1.59E-41
GO:0034470	ncRNA processing	3.98E-38
GO:0000819	sister chromatid segregation	5.71E-38
GO:0140014	mitotic nuclear division	2.86E-37
GO:0042254	ribosome biogenesis	6.54E-37
GO:0000280	nuclear division	1.50E-36
GO:0000070	mitotic sister chromatid segregation	6.10E-35
GO:0098813	nuclear chromosome segregation	8.53E-35
GO:0006310	DNA recombination	1.27E-32
GO:0006302	double-strand break repair	2.25E-31
GO:0051169	nuclear transport	9.64E-29
GO:0006415	translational termination	2.12E-28
GO:0006913	nucleocytoplasmic transport	2.51E-27
GO:0000723	telomere maintenance	3.00E-27
GO:0000375	RNA splicing, via transesterification reactions	6.45E-27
GO:0070125	mitochondrial translational elongation	6.45E-27

Supplementary Table 5B Top 20 GO dowregulated pathways

	**Description**	**qvalue**
GO:0045229	external encapsulating structure organization	1.39E-17
GO:0043062	extracellular structure organization	1.39E-17
GO:0030198	extracellular matrix organization	1.87E-17
GO:0031589	cell-substrate adhesion	1.14E-14
GO:0098742	cell-cell adhesion via plasma-membrane adhesion molecules	3.41E-13
GO:0003012	muscle system process	6.60E-12
GO:0034329	cell junction assembly	8.87E-12
GO:0060485	mesenchyme development	1.20E-11
GO:0006936	muscle contraction	3.24E-11

GO:0007409	axonogenesis	3.24E-11
GO:1903522	regulation of blood circulation	4.48E-11
GO:0048762	mesenchymal cell differentiation	9.45E-11
GO:0018212	peptidyl-tyrosine modification	2.18E-10
GO:0007411	axon guidance	2.49E-10
GO:0097485	neuron projection guidance	3.01E-10
GO:0008016	regulation of heart contraction	3.90E-10
GO:0002576	platelet degranulation	4.11E-10
GO:0018108	peptidyl-tyrosine phosphorylation	4.11E-10
GO:0001503	ossification	4.14E-10
GO:0008217	regulation of blood pressure	4.52E-10

Supplemental Figure Legends:

Supplementary Figure 1. MNX1-dependent viability of GDM-1. A. Representative Western blot targeting MNX1 (top) and relative MNX1 protein quantification after knockdown (bottom). **B.** Knockdown of MNX1 with sh76 and sh82 reduces GDM-1 viability. **C.** Higher sensitivity of GDM-1 to DAC than MNX1 non-expressing AML cell lines HL-60, OCI-AML-3, and MOLM-13.

Supplementary Figure 2. Upregulation of *MNX1*-targeting miR-200a-3p upon DAC treatment. A. Relative expression levels of miR-381-3p, miR-410-3p, miR-200a-3p and miR-3180-5p in DAC-treated GDM-1. **B.** *MNX1* wildtype and mutated (MUT) 3'UTR targeted by miR-200a-3p. **C.** Schematic representation of the genomic region of miR-200a-3p and the upstream CpG-island including the transcription start site (TSS). Reduced DNA methylation levels in the CpG island (chr1: 1096414-1124603) near the miR-200a-3p TSS after DAC treatment (green dots) as compared to untreated (blue dots). **D.** Representative Western Blot targeting MNX1after overexpression of miR-381-3p, miR-410-3p and miR-200a-3p mimics. **E.** quantification of Westerrn blot in D.
